# How do organisational configuration and context influence the quantity and quality of NHS services provided by English community pharmacies? A qualitative investigation

**DOI:** 10.1371/journal.pone.0204304

**Published:** 2018-09-20

**Authors:** Sally Jacobs, Tom Fegan, Fay Bradley, Devina Halsall, Mark Hann, Ellen I. Schafheutle

**Affiliations:** 1 Centre for Pharmacy Workforce Studies, Division of Pharmacy and Optometry, Faculty of Biology, Medicine and Health, The University of Manchester, Manchester, United Kingdom; 2 Centre for Biostatistics, Division of Population Health, Health Services Research and Primary Care, Faculty of Biology, Medicine and Health, The University of Manchester, Manchester, United Kingdom; Brown University, UNITED STATES

## Abstract

Community pharmacies are expanding their role into medicines-related healthcare and public health services, previously the domain of physicians and nurses, driven by policies to improve healthcare access for patients and to address problems of increasing demands and rising costs in primary and urgent care services. Understanding the organisational context into which this expansion is taking place is necessary given concerns over the extent to which pharmacies prioritise service volume over the quality of service provision. As part of a larger programme of work, this paper aims to explore stakeholder perceptions of the organisational and extra-organisational factors associated with service quality and quantity in community pharmacy as an established exemplar of private sector organisations providing publicly-funded healthcare. With ethics committee approval, forty semi-structured interviews were conducted with service commissioners, superintendent and front-line pharmacists, purposively selected from across nine geographical areas and a range of community pharmacy organisational types in England. Interviews were audio-recorded, transcribed verbatim and thematically analysed. Findings highlight the perceived importance of appropriate staffing and skill-mix for promoting service quantity and quality in community pharmacy. Organisational cultures which supported team development were viewed as facilitatory whereas those prioritising business targets over service quality seen to be inhibitive. Older local populations and low patient expectations were thought to limit service uptake as was poor integration with wider primary care services. The contractual framework and commissioning processes were also seen as a barrier to increasing service quality, quantity and integration in this sector. These findings suggest that healthcare administrations should take account of organisational and extra-organisational drivers and barriers when commissioning services from private sector providers such as community pharmacies to ensure that the quality of service provision is incentivised in addition to service quantity. Additionally, collaborative working should be encouraged through integrated commissioning mechanisms.

## Introduction

Concerns over the increasing demands on primary and urgent care services, and the rising costs of healthcare, have driven policymakers to capitalise on the potential of community pharmacies to provide medicines-related and public health services beyond medicines supply. This builds on the concept of ‘pharmaceutical care’[1] whereby community pharmacists utilise their increasingly clinical training and skills to deliver services such as medication reviews, minor ailments services, support for the self-care of long-term conditions and healthy lifestyle services (e.g. smoking cessation, weight management).

In the United Kingdom (UK), the solution that pharmacy may offer to the financial and workload problems facing the National Health Service (NHS) has led to the introduction of healthcare policies which advocate closer involvement of clinical pharmacists and community pharmacy in the organisation and delivery of primary healthcare services.[2, 3] In England, this includes the introduction of a national scheme to employ pharmacists in general practices,[4] an integration fund to support collaborative working with community pharmacies[5] and the creation of new models of care including multispecialty community providers which will integrate primary and community providers, including pharmacy, to serve and improve the health of local populations.[3] This echoes findings published in the Royal Pharmaceutical Society commission on future models of care, *Now or Never*,[6] which called for “a national primary care strategy that embraces the potential of pharmacy alongside that of general practice and nursing, and bold and imaginative commissioning that supports new models of integrated care.” (p47)

However, for the expansion of community pharmacy’s contribution to be a success, a greater understanding of its organisational context is required. Community pharmacies range from single-handed ‘independent’ pharmacies to large national or multi-national chains or ‘multiples’ and supermarkets. They are for-profit organisations delivering medicines-related healthcare alongside the sale of health and non-health related services and products. They are somewhat unusual amongst healthcare providers in that they depend upon income from a range of sources, both retail and healthcare, and are thus subject to business *and* healthcare policy drivers, regulations and pressures. The expansion of healthcare and other public service provision into the private sector has been increasing across Europe and other developed countries, driven by a desire to increase patient choice and access to services whilst improving efficiency.[7] However, concerns have been raised over the quality and safety of patient care[8] and the motivations of managers[9] in private sector organisations where there is a need to balance delivery of healthcare with generation of profit.

In England, community pharmacy dispensing, medicines-related healthcare and public health services are provided under contract to the publicly-funded NHS (responsible for commissioning the vast majority of primary, secondary and tertiary healthcare services both nationally (NHS England) and locally (clinical commissioning groups; CCGs)) and local authorities (responsible for commissioning public health services at a local level), primarily on a fee-for-service basis. The current contractual framework for community pharmacy was introduced in 2005 to place greater emphasis on the delivery of extended services alongside more traditional supply functions. It introduced three service tiers: nationally commissioned *essential* (e.g. dispensing) and *advanced* (e.g. medicines use reviews (MURs)) services, and *locally commissioned* (medicines-related healthcare and public health) services. However, the associated rise in the *quantity* (range and volume) of services provided has been associated with a worrying increase in pharmacists’ workload[10] and claims that excessive pressure to meet contractually-incentivised business targets, particularly in the larger pharmacy chains, risks compromising service *quality*.[11, 12]

As part of a wider, mixed-methods study of clinical productivity in English community pharmacies published in full elsewhere,[13] this qualitative investigation sought to explore the organisational and extra-organisational factors associated with the quantity (range and volume) and quality of services provided.

## Method

This paper presents findings from a series of semi-structured face-to-face and telephone interviews conducted between November 2014 and April 2015.

Research ethics approval was obtained from the UK National Research Ethics Service (13/WM/0137) and endorsed by the University of Manchester Research Ethics Committee (13025).

### Setting

The study was conducted across nine socio-economically diverse geographical areas of England, purposively selected to cover locations in the north and south, rural, urban and suburban areas and differing degrees of deprivation. All community pharmacies in these primary care administrative areas, bar those owned by four non-participating national chains, were invited to take part in a survey of organisational characteristics. Of those responding (227/800 (34.6%)), 39 (from a stratified random sample) participated in a patient survey, and all interviewees were drawn from these 39 pharmacies and the NHS commissioning bodies (CCGs and NHS England area teams) operating across the nine study sites.

### Sample

Interviewees were selected purposively to include those directly involved in the commissioning and provision of community pharmacy services: at least one pharmacy commissioner (from NHS England and clinical commissioning groups (CCGs)) and a cross-section of frontline and superintendent (appointed to represent the pharmaceutical aspects of a retail pharmacy business) pharmacists from pharmacies of differing ownership types (independent, small/medium and large chains) from each geographical area.

### Recruitment

Participants were contacted by email, followed up by telephone to discuss the study, before obtaining written informed consent to participate. Where individuals chose not to participate, attempts were made to recruit a similar replacement (by study area; pharmacy ownership type) until all available options were exhausted.

### Interview content and process

Interviews, conducted by SJ, TF and FB (none of whom had pre-existing relationships with any participant), took a broadly phenomenological approach,[14] with topic guides specific to each stakeholder group (commissioner, pharmacist (S1 File), superintendent pharmacist) developed from the study aims and research literature. Lines of questioning explored definitions of quality, the relationship between the quantity and quality of service provision in community pharmacies, opportunities and barriers to maximising clinical productivity and the mechanisms by which different organisational characteristics may help or hinder this objective. A prompt sheet listing the organisational factors of interest was sent in advance (S2 File).

All interviews were audio recorded, with consent, and transcribed verbatim.

### Data analysis

Interview data were thematically analysed, supported by NVivo 10. A framework approach[15] was adopted, with analysis involving five steps: familiarisation; developing a thematic framework; indexing; charting; and mapping and interpretation. Two researchers (TF and FB) undertook the first four steps collaboratively, developing the thematic framework through independent familiarisation with different sets of interview transcripts, followed by close discussion and agreement of identified themes. Where consensus could not be reached, a third researcher (SJ) was brought in. The agreed thematic framework was applied and charted by TF and FB and the final stage of mapping and interpretation was undertaken by SJ. The face validity of the findings was examined during a stakeholder workshop held in July 2015 attended by service commissioners, community pharmacy representatives and service users.

## Results

### Interviews conducted

Forty interviews were conducted face-to-face (21) or by telephone (19), lasting 33–97 minutes. Ten participants were service commissioners and 30 were pharmacists/superintendent pharmacists (Table 1). No substantive differences were found between the data elicited from face-to-face and telephone interviews.

**Table 1:** Study participants.

NHS service commissioners (n = 10)	Pharmacists (n = 30)
NHS England area teams (5)	Superintendent pharmacists (5) • Small chains (1) • Large multiples/supermarkets (4)
Clinical commissioning groups (5)	Dual superintendent/patient-facing roles (6) • All small chains/independent pharmacies
	Patient-facing pharmacists (19) • Large multiples/supermarkets (9) • Small/medium chains (6) • Independent pharmacies (4)

The findings from pharmacist and service commissioner interviews are presented together under the main themes: definitions of quality, organisational and extra-organisations characteristics associated with the quality and quantity of service delivery.

### Definitions of quality

Interviewees were asked how they would define service quality in community pharmacy, specifically in relation to dispensing services and MURs. MURs were one of the advanced services introduced in 2005 and involve a pharmacist consultation to improve a patient’s understanding of and adherence to their medicines.

For dispensing, speed and accuracy were the most commonly cited elements of service quality. Most frontline and superintendent pharmacists believed that speed of dispensing, and thus limited waiting time, was valued by patients above anything else. However, for pharmacists themselves, and for many service commissioners, accuracy was paramount. Additionally, clinical aspects were considered by a number of pharmacists and commissioners to be an important element of quality either through counselling or the clinical check.

“*I would want a really strong cognitive element at the beginning in terms of the clinical check so people really thinking about what they’re doing rather than necessarily looking at prescriptions thinking “have we got this in stock?” Thinking critically about that prescription and at the end of the process making sure that patients know what they’re taking and why they’re taking it and recognising that there is somebody that they can call upon should they have any problems. So there’s the whole of the patient counselling piece at the end which I think is often forgotten about in the spirit of getting prescriptions done as quickly as possible to meet customer demand*.” *[Superintendent Pharmacist 3]*

Less often mentioned but considered to be additional domains of dispensing quality were the importance of maintaining stock levels, to prevent patients having to wait or return, and offering good customer service.

In relation to the MUR service, quality was defined by most interviewees in terms of outcomes for patients, e.g. increasing knowledge and understanding of medications, improving adherence, addressing side-effects, improving clinical outcomes and quality of life, and providing reassurance.

“*I only find them [MURs] worthwhile when you have an outcome at the end of it. So if, say for example, they've been experiencing a side-effect and they haven't linked it to a particular drug, or you've actually offered something more to them to either improve their regime, or to resolve a problem that they've been having, or there's been some kind of positive outcome where they either feel more reassured about taking the medication or there's been some kind of change, or something else that you have to offer which has helped in any kind of way—then I find them worthwhile*.” *[Pharmacist 38]*

Targeting of MURs to patients most likely to benefit, e.g. those taking several medicines or people with certain long-term conditions including respiratory and cardiovascular disease, was mentioned by several interviewees as an important element of quality.

Many perceived that taking a patient-centred approach also contributed to a high quality MUR, utilising communication skills and tailoring the consultation to a patient’s needs. Where speed was seen an important element of quality dispensing, allowing adequate time was perceived as important for MURs to be of benefit. Less frequently mentioned were the provision of healthy living advice, integration with other pharmacy or general practitioner (GP) services and for the patient to be engaged with the process.

### Organisational characteristics associated with the quality and quantity of service delivery

#### Staffing and skill-mix

All pharmacists/ superintendent pharmacists and most service commissioners mentioned staffing and skill-mix as an important, possibly the most important, organisational factor influencing the quantity and quality of community pharmacy services. This related to overall staffing levels, skill-mix, training, teamwork, delegation and continuity of staff.

Insufficient staffing levels were often reported with implications for service quality (increased waiting times, decreased clinical input, increased risk of dispensing errors) and service quantity (dispensing prioritised over MURs and other services).

“*I think the main thing is staffing. It’s because margins are being cut and employers, owners, can’t afford to employ more staff, so everybody’s under pressure and the things that go are the services. You can’t take ten minutes out of your day to do an MUR if you’ve got piles of prescriptions that need checking*.” *[Pharmacist 41]*

For many commissioners, continuity of pharmacy staff and turnover was perceived as a particular problem to the reliability and quality of service delivery.

“*With the pharmacy services, it’s often the individual pharmacist that’s either keen or not keen. And so we very often have the experience of phoning or going into a pharmacy and saying, “Oh hello, can I ask you if you’re providing the minor ailments service today?” for example. And they’ll say, “Oh no, we’ve only got a locum on today”, or, “Oh, well, we used to but our regular manager’s gone off on maternity leave and we’re covered by temporary staff now*”” *[Commissioner 1]*

More commonly discussed by pharmacist and superintendent interviewees than staffing levels was the skill-mix of the pharmacy team and its influence on the quality and quantity of pharmacy services. A key enabling factor was the support of a trusted and competent team, each trained to an appropriate level, to free up the pharmacist to be more patient-facing and provide more clinical and higher quality services, spending more time with patients. It was suggested that this would be more likely to improve patient outcomes (e.g. adherence) and reduce waste.

Many interviewees (both pharmacist and commissioner) emphasised the role of accuracy checking technicians (ACTs; pharmacy technician or other member of the pharmacy team qualified to conduct the final accuracy check on dispensed prescriptions) in the dispensing process as a successful way of freeing up pharmacists’ time for clinical services, extending the range offered, volume delivered and also the quality of those services (e.g. time spent with a patient for an MUR).

“*I think a big thing is if you can get an ACT…it frees up your time incredibly. You have a dispenser and an ACT, you’ve got nothing to worry on the dispensing side, then you’ll become fully patient-focused…you can offer other services, diabetes screening and blood pressure monitoring*.” *[Pharmacist 15]*

Others suggested that the ability to employ a second pharmacist (most pharmacies operate with only one pharmacist) would provide the ideal solution to pharmacies looking to expand their range of services. However, the financial barriers to this were recognised.

“*I would really like to see two pharmacists in every premises, so that they could cover for each other and provide the clinical services that I would want to see from community pharmacy. I don’t think that’s going to happen until it’s part of the national contract. And perhaps the national contract has to move away from an item-based fee basis, onto more of a population, wellbeing basis*.” *[Commissioner 10]*

#### Workload and its management

Most interviewees perceived that workload and time constraints were a ubiquitous barrier to both the range and quality of services provided. Several factors were thought to have contributed to increasing demands on pharmacists’ time including: increasing (and fluctuating) dispensing volumes; reduced staffing; the growing range of pharmacist-led services; an increasing regulatory and administrative burden; and stock shortages.

“*It’s going to get to tipping point, I think, where there’s so much pressure on the teams. And, not just the pharmacist time, the dispensers I’ve worked with, I can’t say enough good things about them…sometimes they’re in the back and they’re in tears because they’re that stretched. They know they’re working at absolute capacity and it’s still not enough, and they still can’t get through the work*.” *[Pharmacist 29]*

High dispensing volumes limited both the quality and quantity of services delivered in a number of ways. It reduced the time available for counselling and follow-up of patients, increased waiting times and threatened accuracy. Because of the reactive nature of dispensing, this was often prioritised over other services, reducing not only service volume but also quality, e.g. by only offering MURs to less complex cases or reducing the time spent in consultation.

A number of workload management strategies were described by pharmacist interviewees. These included: appointment systems for MURs and other services, mechanisms for handling dispensing of repeat prescriptions, pharmacy level procedures, and the use of technology. Service commissioners endorsed the need for pharmacies to adopt better workload management systems.

“*Everybody gets lulls in the day…so it might be that if you're quiet from two 'til three before the sort of late afternoon surge kicks in, then it might be that if, you were offering …a pre-bookable service, like health checks…you would say to your staff “well, look, that's a really good slot.” That's what the companies are good at doing…they literally look at the till receipts, so they can see the surges in business…and the dispensing flow and everything, and they target the services around those dips in walk-ins*.” *[Commissioner 3]*

Whilst some pharmacists were able to use an appointments system for managing competing workloads, others felt that this was not workable because of the unpredictable nature of their walk-in business. A number of methods of organising the more predictable workload associated with repeat prescription dispensing were also described by pharmacist interviewees, including the use of collection services, off-site dispensing and pharmacy-level procedures for managing dispensing workflow, all of which helped some pharmacies free up time to maximise both the quantity and quality of service provision.

#### Pharmacy ownership and organisational culture

A number of opportunities and barriers to service quality and quantity were identified by interviewees in relation to the type of organisation a pharmacy belonged to (e.g. large multiple, supermarket, smaller chains and independents). Many interviews highlighted the central role of organisational culture, or “the way we do things around here,”[16] describing it in terms of the extent to which business targets (or quantity) were prioritised over service quality, the value the organisation (head office, the superintendent pharmacist or pharmacy owner) placed on investing in staffing and skill-mix, and management style and structure.

Most pharmacists interviewed reported the existence of service targets to help maximise service volume. Some recognised that targets could be helpful in ensuring that a range of services was provided. However, where the culture of the organisation was one where the pressure to meet targets was perceived as excessive or as prioritising profit over meeting patient needs, this was viewed as detrimental to service quality.

“*Everybody I know thinks it's quantity, not quality. If you don't set targets, maybe nothing will ever get done. But setting targets just creates rubbish; you just end up with rubbish being done to just earn some money*.” *[Pharmacist 34]*

Although it was recognised that all pharmacy organisations placed a certain degree of pressure on pharmacists to maximise productivity, a number of interviewees perceived this pressure to be excessive in some large multiples. Reported managerial strategies to enforce targets varied from collaborative, supportive and encouraging to autocratic, discouraging and humiliating. There were reports of daily pressure from some area managers (responsible for overseeing pharmacy branches in a locality) to hit targets and, in extreme cases, bullying.

Conversely, in pharmacies where the culture supported investment in staffing, skill-mix and training, benefits were seen in terms of both the quality and quantity of services provided.

“*Skill mix we very much believe in, that’s why we have ACTs, and we train staff up as much as we can, as long as we feel that they’re going to be able to practise their new role and use those skills. We pretty much believe that the majority of our staff…should be at least pharmacy assistant trained. And, we’re quite happy to use our ACTs, such to enable pharmacists to carry out the services, and that’s the philosophy of the company*.” *[Superintendent Pharmacist 3]*

Some interviewees perceived that, staffing levels were not increasing in line with increasing workloads and, in some cases, were being pared back, particularly in large multiples. It was felt that, in such cases, the pharmacy team’s capacity to provide additional services was compromised, and patient safety might be at risk. Independent pharmacies were viewed by some to be more willing to increase staffing levels which helped engender a better patient experience.

“*[Patients] said, we’re treated with courtesy, we’re treated with respect, we’re dealt with promptly and it’s all the sort of things that the patients value. They’ve always got time for you. There’s always a pharmacist there, so I can quickly talk to them…despite the sheer volume of prescriptions that they deal with. Definitely the independents have that ability because they always have more staff, whereas the multiples are very much it’s down to that n^th^ degree of the staffing*.” *[Commissioner 1]*

Yet others felt that a better quality of service could be delivered by large multiples because of investment in training for pharmacists and staff. A small number also perceived that this was augmented by the level of support for the pharmacy team from head office.

The role of management style and structure in supporting service delivery was also highlighted in relation to the extant culture of the organisation. For example, having a non-pharmacist area manager, who could be perceived as unreceptive to pharmacists’ problems, was sometimes seen as detrimental to productivity; having a non-pharmacist store manager, conversely, could be seen to be beneficial by taking on pharmacy management and administrative tasks and freeing up pharmacists’ time.

### Extra-organisational factors associated with the quality and quantity of service delivery

#### Patient and population characteristics and expectations

The demographic of the local population was perceived to influence the range and volume of services community pharmacies could offer. For example, a number of pharmacists highlighted that being situated in an area with a large proportion of older residents limited both the number of MURs they could conduct and the range of other services they could offer. Although it was recognised that domiciliary MURs could be conducted, regulatory and organisational barriers often meant that they were not.

“*… unfortunately, those people that…most need our services, are quite often housebound patients that you never see in the pharmacy…things like MURs are completely useless, because you’re not seeing the patient*.” *[Pharmacist 3]*

There was also the perception that older people were more likely to visit their GP for services that could otherwise be provided by the pharmacy (e.g. flu vaccinations; glucose monitoring) either through preference or service restriction.

A number of pharmacists and commissioners perceived that public perceptions and expectations of community pharmacy–of the services available and pharmacists’ roles–could influence both the volume and quality of services. Although it had been ten years since the introduction of MURs and an extended range of other medicines optimisation and public health services from community pharmacies, public perception that pharmacies are only there to dispense prescriptions remained an important perceived barrier.

#### Community pharmacy–general practice relationships

The strength of a pharmacy’s relationship with its local GP surgery(ies) was cited by a large number of pharmacists, superintendents and all commissioners as an important factor influencing the quality and quantity of community pharmacy services. Positive relationships were seen to help nurture interdisciplinary practice, foster closer working around patients, increase effective signposting and improving communication.

“*I also think that good communications and relationships…makes a huge, huge difference to the working lives of pharmacists and GPs […] So the wider working relationships are as important as the staff to underpin that service*.” *[Commissioner 5]*

Many pharmacists believed that good relationships with local GP surgeries enabled prompt resolution of issues, for example problems with prescriptions, clinical interventions and stock shortages. This had a positive effect on service quality, with the opposite effect where poorer relationships existed.

“*Sometime we have problems, you know, with the GP …the receptionist, but I suppose that everybody’s got the same problem. The way you’re to trying to help a situation, they will hinder it basically. That causes a massive problem with the flow of things*.” *[Pharmacist 28]*

A number of pharmacists felt that positive relationships encouraged GPs to initiate contact with pharmacists, for example, to request advice and follow-up recommendations from MURs. Indeed, some perceived that effective working relationships were optimised where local GPs had developed trust in the pharmacist. Good working relationships could therefore help increase pharmacy service volume and range through referral or signposting of patients. Commissioners also spoke about the importance of GP endorsement of pharmacy services, to increase patient uptake.

“*If a GP said to a patient, “Oh, by the way the pharmacy down the road offers this really good service called an MUR and I think it’s really important that you go and talk to them about your inhaler technique”, the patient is far more likely to do that… if you get that endorsement from GPs around pharmacy services I think that carries a lot of weight with patients*”. *[Commissioner 6]*

However, for some pharmacists, referral or signposting to a pharmacy was believed to be, at best, selective for some services, with competition between pharmacies and general practices to provide some services encouraging silo behaviours.

“*…pharmacy/GP integration is something that needs to be focused upon…The problem is… they’re two competing businesses…there are certain cross-over areas, such as flu vaccinations and things like that….If we work together…pharmacists can help the GPs to achieve their targets…[and] at the same time…also enable the pharmacist to provide another service*.” *[Pharmacist 3]*

#### Commissioning and contractual arrangements

As community pharmacies are private businesses required to make a profit, many of the pharmacists and superintendents interviewed perceived that the level of remuneration for services was insufficient for investment in staffing and infrastructure (e.g. consultation rooms/IT) necessary to be able to deliver these services.

“*To enable you to do MURs you have to invest in a consultation room…Then you need…a second PMR [patient medication record] system to use in that room. In order to free the pharmacist up you need a team of better trained counter staff and dispensary assistants. When you add up all those costs you would be much better off to forget the MURs … the economics don't work*.” *[Superintendent Pharmacist 4]*

Other contract-related issues seen as having a detrimental effect on clinical productivity included the short commissioning cycle operated by NHS commissioners, seen as a barrier to investment in staffing levels and training.

“*[Pharmacies] don’t know whether it’s worthwhile doing [services] because two years down the line they mightn’t get paid for it, or they might be expected to do it without the same reimbursement*.” *[Pharmacist 35]*

Some interviewees suggested changing the basis of remuneration from fee-for-service to service quality or outcomes, e.g. having a pharmacy quality and outcomes framework (QOF) in line with general practice. It was suggested that this could incentivise an increase in the quality of community pharmacy services, and also alleviate pressure on general practice workloads.

“*A QOF for pharmacy would be good… [The pharmacy contract] doesn’t appear to be aligned particularly well to the GP contract so we’ve not necessarily got two professions working in the best interests of patients. I think a root and branch review of the pharmacy contract would be a good start, and start to recognise the clinical value that pharmacists add through their interaction with patients*” *[Superintendent Pharmacist 3]*

Lastly, at the time this study was conducted, the NHS in England had just undergone a substantial re-organisation leading to the fragmentation of commissioning responsibilities for community pharmacies between NHS England (essential and advanced services), CCGs (locally commissioned medicines-related healthcare services) and local authorities (locally commissioned public health services). This not only caused confusion amongst many pharmacy interviewees and increased bureaucratic processes but was also associated with some service decommissioning. This reduction in service provision, by reducing service accessibility to patients, was also seen as lowering the quality of NHS services.

*“Most of the public health initiative type services*, *have gone onto the local authority tendering format which has put a much increased burden bureaucratically on community pharmacies…there are a number of pharmacies that have said*,*…“That’s too much like hard work to complete all of that paperwork …*. *Actually I’m busy enough as it is; I’m not going to bother*.*”… That leads to poorer quality of service because there’ll probably be fewer pharmacies delivering certain services and therefore you haven’t got the accessibility for the service that was previously available*.*" [Superintendent Pharmacist 5]*

## Discussion

The findings presented here are based on interviews with community pharmacists, superintendents and commissioners as part of a larger study into clinical productivity in community pharmacy. These findings have highlighted the implications of increasing workloads in community pharmacy and the importance of adequate staffing and skill-mix to support service expansion alongside increasing dispensing volumes. Organisational culture plays a central role in determining productivity in relation to the extent to which it engenders staff and team development or prioritises business targets over service quality. Extra-organisationally, the characteristics of the local population and patient expectations, as well as working relationships with local GPs and (lack of) integration with the services they provide, can limit the uptake of services. The influence of the nature of the contractual framework and commissioning processes on the quantity and quality of service provision has also been highlighted. Although not all of these issues are new, this paper provides the first comprehensive insight into the organisational and extra-organisational factors associated with the quantity (volume and range) and quality of services provided by community pharmacies and an exploration of their mechanisms of action. These findings thus offer explanatory insights for the outcomes of a linked quantitative investigation, which concluded that whilst a pharmacy’s dispensing volume was positively associated with local population need, the volume of MURs delivered were driven more by corporate ownership and that, whilst levels of staffing and skill-mix were associated with dispensing volume, they were not associated with levels of MUR provision.[13, 17]

This study has some important limitations. Whilst socio-economically diverse, the geographical coverage of study sites was limited to those selected for the wider study. As with most qualitative studies, the findings cannot be said to be generalisable to the wider population. Nonetheless, the sample was purposively selected to cover the full range of pharmacy ownership types and the sample size was sufficient for the requirements of data saturation to be met. Furthermore, our sample provided perspectives from front-line community pharmacists, superintendents and commissioners.

The problem of increasing workloads in UK community pharmacy since the introduction of the 2005 contractual framework has been well documented, particularly in relation to the impact on pharmacists’ well-being and the potential detriment to patient safety.[10, 18] This problem is not unique to the UK, with workload implications of pharmacists’ role expansion and workforce shortages reported as having an impact on levels of job stress and commitment, patient safety and the adoption of new services in several countries, particularly the United States (US).[19–22] The central importance of adequate staffing, appropriate skill-mix and teamwork in supporting this increasing workload and the quality and safety of community pharmacy services was emphasised throughout these interviews. In the absence of any growth in funding for community pharmacy services, or indeed the threat of funding cuts[23], pharmacies will need to rethink how they can remain competitive, viable and make increasing contributions to medicines supply and public health services. Whilst staffing levels are being cut, this could become unmanageable in light of already excessive workload pressures and job stress amongst community pharmacy staff. It will therefore be necessary for community pharmacy organisations to look more closely at skill-mix and opportunities to deploy staff effectively to support the expansion of services. In addition to often a single pharmacist, the community pharmacy team includes pharmacy technicians, medicines counter assistants, dispensing assistants, and accuracy checkers. Although in some European countries (e.g. Denmark, the Netherlands) pharmacy technicians routinely undertake dispensing without pharmacist supervision, in the UK and US pharmacists are required to either undertake or supervise different parts of the process. Some research exists which supports the expansion of the roles of pharmacy technicians and other support staff in community pharmacy[24, 25] which would help free up pharmacists’ time for clinical roles.

Community pharmacies are long-standing private sector organisations,[26] and the expansion of services provided by them offers a good exemplar to explore the implications of a more widespread move towards a mixed economy of healthcare.[27] There may be a risk in some organisations for an organisational culture to prevail where profits are prioritised over patient care, and service quantity over quality. Indeed, the quantitative research linked to the interviews reported here suggests that the delivery of some community pharmacy services may be driven more by organisational factors than local population need.[13, 17] There is a trend towards the corporatisation of healthcare (the use of market mechanisms, growth in for-profit provision, and increasing size of provider organisations), in pharmacy[28] and also in primary care provision more widely.[29] These findings may therefore have implications for the way in which such services are commissioned, to ensure that service quality is incentivised alongside quality.

A contract which offers remuneration only on a fee-for-service basis appears, from these findings, to incentivise quantity over quality. Moreover, the short term nature of local service commissioning does little to encourage growth. Healthcare administrations need to better understand the nature of private sector provider organisations when implementing change, so that the commissioning of publicly-funded healthcare services from private sector organisations, such as community pharmacies, incentivises quality as well as quantity of service provision by offering remuneration on the basis of process and outcome quality measures, although these remain to be defined. Moreover, commissioning and remuneration processes should be integrated within healthcare systems to encourage collaborative working, in this case between community pharmacies and general practice. Rewarding joint working through integrated commissioning could encourage the signposting of patients to community pharmacy services by GPs, for example, which is known to encourage service uptake.[30]

## Conclusion

Through identifying the key drivers and barriers to service expansion in community pharmacy, this study offers valuable insights both for community pharmacy organisations and for service commissioners. Exploration of the organisational context has highlighted for pharmacies the importance of staffing and skill-mix and engendering a supportive, team-building culture. For healthcare administrations, it has suggested a need to better understand the organisational culture within private sector providers to ensure the quality of services commissioned outwith the public domain. Moreover, the importance of extra-organisational factors such as relationships with local general practices and contractual arrangements has implications for future contractual and remuneration arrangements.

## Supporting information

S1 File Pharmacist interview topic guide. (DOCX)

S2 File Interview prompt sheet. (DOCX)
