# Supplementary material for: How do organisational configuration and context influence the quantity and quality of NHS services provided by English community pharmacies? A qualitative investigation
Source: PLoS One. 2018 Sep 20;13(9):e0204304. doi: 10.1371/journal.pone.0204304 (PMC6147574; doi:10.1371/journal.pone.0204304)
Supplement: S1 File — (DOCX) [file pone.0204304.s001.docx]

**Pharmacist interview topic guide**

**Background**

First of all I would like to get a bit of background information about you.

- What position do you currently hold?
- How long have you been in your current post?
- Have you had any pharmacy jobs before this one? [probe: previous jobs/ experience/ qualifications]
- Could you briefly describe your current roles and responsibilities?

We also need a bit of information about your pharmacy [cross-check with survey data where possible and KEEP BRIEF].

- How would you describe the type of pharmacy you work for? [Probe: multiple, supermarket, small/medium chain, independent? How many stores?]
- In terms of how the pharmacy is managed, is the pharmacy manager a pharmacist? (or are managers pharmacy technicians or non-pharmacy professional?) And who manages the main pharmacist? Are they themselves a pharmacist?
- What staff do you have working here? [probe: levels, skill-mix (who does what?) and differences throughout the day/week] – KEEP BRIEF
- Roughly how many items do you usually dispense in a day? How many MURs would you normally conduct in a week/month? What other NHS services do you offer and what is the take-up of these? What about private services? How does the workload of the pharmacy vary throughout the day/week? – KEEP BRIEF

**Defining clinical productivity**

This is a study of clinical productivity in community pharmacy and what pharmacies and service commissioners can do to maximise this.

- Is ‘clinical productivity’ a term that is used much in community pharmacy? What does the term mean to you (if anything)?

We are looking at clinical productivity in terms of both the *quality* and *quantity* of service provision. The *quality* of the pharmacy service is associated with individual patient benefit. The *quantity* is also important because the more patients that receive a high *quality* service, the greater the effect the service will have on public health. Conversely, high *volumes* of low *quality* services have a *potential* cost both to the NHS and to patients/customers.

**Quality and quantity of pharmacy service provision**

1. **Quantity**

Thinking about the quantity of services provided by your pharmacy – and by quantity I mean both the number or range, and volume of different services provided by your pharmacy (essential, advanced and locally commissioned) – would you say that this level of activity is high (above average), about average, or low?

- What aspects of the way your pharmacy operates do you think helps you to maximise this level of service delivery? [probe which organisational factors and how they help– PROMPT SHEET]
- What aspects of the way your pharmacy operates do you think gets in the way of you maximising the quantity of the different services you deliver? [probe which organisational factors and how they get in the way – PROMPT SHEET]

1. **Quality**

I would now like to ask you about the quality of the services your pharmacy provides.

Thinking first of all about dispensing, what for you characterises a ‘high quality’ dispensing service provided by a pharmacy?

- What does a high quality dispensing service look like? [probe: pharmacy team perspective, patient perspective]

How often do you feel the dispensing service provided by your pharmacy achieves this level of quality? [probe: level of quality actually achieved, how this deviates from ‘high quality’]

- Thinking about how this pharmacy operates, what aspects do you think help you to provide a high quality dispensing service? [probe which organisational factors and how they help – PROMPT SHEET]
- What aspects do you think get in the way of you providing a high quality dispensing service? [probe which organisational factors and how they get in the way – PROMPT SHEET]

Thinking now about MURs, what for you characterises a ‘high quality’ service provided by a pharmacy?

- What does a high quality MUR look like? [probe: pharmacy team perspective, patient perspective]

How often do you feel that the MURs provided by your pharmacy achieve this level of quality? [probe: level of quality actually achieved, how this deviates from ‘high quality’]

- Thinking about how this pharmacy operates, what aspects do you think help you to provide high quality MURs? [probe which organisational factors and how they help – PROMPT SHEET]
- What aspects do you think get in the way of you providing high quality MURs? [probe which organisational factors and how they get in the way – PROMPT SHEET]

**Changes in clinical productivity**

Have you been aware of any changes in the quantity (range and/or volume) of services delivered by this pharmacy over the past 5 years/ since you’ve worked here?

- [if yes] Could you describe these changes?
- What do you think have been the main causes of these increases/decreases?
- Has your organisation made any changes to the way it operates to adapt to these changes in levels of service delivery? [probe organisational factors – PROMPT SHEET]
- [If yes] How successful do you feel these changes have been?

Have you been aware of any changes in the quality of service delivery in this pharmacy over the past 5 years/ since you’ve worked here?

- [if yes] Could you describe these changes?
- What do you think have been the main causes of this increase/decrease in quality?
- Are there any aspects of the way your pharmacy operates which you think have contributed to these changes in quality? [probe organisational factors – PROMPT SHEET]

**Maximising clinical productivity**

The NHS is continually seeking to maximise clinical productivity to get “more bang for its bucks.” We also know that, in general, dispensing volumes are rising and pharmacies are increasingly being commissioned to provide a wider range of services.

To what extent do you think it is possible for pharmacies to continue to provide a greater quantity of services whilst maintaining (or improving) the quality of the service provided?

- What aspects of clinical productivity do you think could be improved?
- What do you think community pharmacies need to be doing to help achieve this? [probe organisational factors – PROMPT SHEET]
- What do you think are the main barriers to improving clinical productivity in community pharmacy? [probe organisational factors – PROMPT SHEET; external factors]
- What do you think the NHS as service commissioners need to be doing to help achieve this in community pharmacy?

**Measuring/ monitoring clinical productivity**

We are also interested in how clinical productivity might be measured and monitored.

How do you know if you are providing high quality services at the appropriate level?

Does your pharmacy formally monitor/ measure clinical productivity (i.e. both the quality and quantity of services provided)?

- [If so] How? [Prompt: audits, patient survey]
- How are the findings then used?
- Do you think this helps to improve clinical productivity in your pharmacy?
- [If not] Do you think it should?
- How?
- Do you think this would help to improve clinical productivity in your pharmacy?

Do service commissioners monitor/ measure clinical productivity in your pharmacy? [Prompt: mystery shoppers, audits, quality-improvement instruments etc.]

- [If so] How? For instance, what is done to measure the *number* of MURs performed and assess the *quality* of this service?
- Do you know how these findings are used?
- Do you think this helps to improve clinical productivity?
- [If not] Do you think they should?
- How?
- Do you think this would help to improve clinical productivity in your pharmacy?

Do you think more should be done to monitor/ measure clinical productivity in community pharmacy?

- [If so] Who do you think should be responsible for monitoring/ measuring clinical productivity in community pharmacy?
- How do you think clinical productivity should be monitored/ measured in community pharmacy?
- What do you think the benefits might be? [probe: to commissioners, to pharmacies, to patients]
- What do you think the drawbacks might be?
- [If not] Why not?

Is there anything else you would like to add about clinical productivity in community pharmacy that we’ve not already covered?
