# Supplementary material for: How do organisational configuration and context influence the quantity and quality of NHS services provided by English community pharmacies? A qualitative investigation
Source: PLoS One. 2018 Sep 20;13(9):e0204304. doi: 10.1371/journal.pone.0204304 (PMC6147574; doi:10.1371/journal.pone.0204304)
Supplement: S2 File — (DOCX) [file pone.0204304.s002.docx]

**Interview prompt sheet**

**Organisational factors under investigation:**

**Location** – geographical and physical

**Ownership** – e.g. independent, chain, supermarket

**Type of contract** – e.g. standard, 100-hour, essential small pharmacy

**Healthy Living Pharmacy status**

**Volume of work** – number, range and volume of different services offered by the pharmacy; how that fluctuates throughout the day, week, year

**Staffing** – number and type of staff in pharmacy; turnover; experience

**Skill-mix** – who does what?

**Use of locums** – frequency and turnover

**Continuity of care for patient** – whether or not patients/customers see the same members of the pharmacy team on each visit

**Management** – structure and style

**Working hours** – opening hours, working hours, shift work

**Organisational culture** (‘how things get done around here’) – the set of implicit or explicit values, beliefs, rules and behaviours which govern how the pharmacy operates

**Safety climate** – the organisational culture in relation to patient safety

**Technology/automation** – use of computers, automated dispensing, etc.

**Pharmacist/GP integration** – working relationship between pharmacy and nearby general practice(s)

**Anything else?**
